# Minimally invasive posterior cervical foraminotomy versus anterior cervical fusion and arthroplasty: Systematic review and updated meta-analysis

**DOI:** 10.1016/j.bas.2024.102852

**Published:** 2024-06-25

**Authors:** Hanmo Fang, Min Cui, Kangcheng Zhao, Yukun Zhang, Xianlin Zeng, Cao Yang, Lin Xie

**Affiliations:** Department of Orthopaedics, Union Hospital, Tongji Medical College, Huazhong University of Science and Technology, 430022, Wuhan, China

**Keywords:** MI-PCF, ACDF, CDA, Complications, Reoperation, Meta-analysis

## Abstract

**Introduction:**

This study is a systematic review and meta-analysis that investigates the efficacy of different surgical methods for treating cervical disc herniation or cervical foraminal stenosis.

**Research question:**

The research aimed to compare the efficacy of Minimally Invasive Posterior Cervical Foraminotomy (MI-PCF) with anterior approaches, namely Anterior Cervical Discectomy and Fusion (ACDF) and Cervical Disc Arthroplasty (CDA).

**Material and methods:**

The study included a comprehensive review of eight articles that compared ACDF and MI-PCF, and four articles that compared CDA to MI-PCF.

**Results:**

The results indicated no significant difference in surgical duration, hospital stay, complication rates, and reoperation rates between MI-PCF and ACDF. However, when comparing CDA with MI-PCF, it was found that CDA had a higher complication rate, while MI-PCF had a higher reoperation rate.

**Discussion and conclusion:**

Despite these findings, the study recommends MI-PCF as the preferred surgical method for cervical radiculopathy, owing to the advancements in minimally invasive techniques. However, these findings are preliminary, and further research with longer follow-up periods and larger sample sizes is necessary to confirm these findings and to further explore the potential advantages and disadvantages of these surgical methods.

## Highlights

• MIS-PCF and ACDF showed no significant advantage in many aspects.
• MI-PCF had a lower complication rate than CDA, showing potential advantage.
• The reoperation rate of MI-PCF was higher than that of CDA.
• Further research is needed to confirm the superiority of MI-PCF.

## 1 Background

Cervical radiculopathy, a common clinical manifestation of cervical spondylosis in clinical practice, is characterized by pain, numbness, and muscle weakness in the upper limbs caused by nerve root compression (Iyer and Kim, 2016). This condition is often secondary to disc herniation in the foraminal region or bony stenosis of the foramen. Surgical treatment should be considered when conservative treatment is ineffective (Van Geest et al., 2014). MI-PCF was proposed as a minimally invasive approach as early as 2000 and has since gained increasing popularity among clinicians for the treatment of cervical radiculopathy (McAnany et al., 2015; Burke; Clark et al., 2011). At present, it is primarily performed in three ways: microscopy with tubular access (Adamson, 2001; Fessler and Khoo, 2002; Hilton, 2007), single-port endoscope (Ruetten et al., 2007), and dual-port endoscope (Park et al., 2017; Wang et al., 2023).

ACDF is the classic surgical approach for the treatment of cervical spondylosis, including myelopathy and radiculopathy. This approach accesses the anterior aspect of the vertebral bodies and the deep intervertebral discs through the intermuscular gap in the anterior pathway, without the need to sever musculature. This approach is less invasive than extensive muscle dissection for posterior surgery. Its typical advantages include a lower incidence of postoperative neck pain and infections. However, the anterior approach also has its complications, including postoperative dysphagia, injury to vital organs such as the esophagus or blood vessels, and even critical complications like anterior cervical hematoma compressing the airway. ACDF, being an anterior fusion surgery, requires internal fixation devices, raising concerns about postoperative non-fusion of pseudarthrosis and adjacent segment degeneration after fusion (Epstein, 2019). Due to fusion, the incidence of adjacent segment degeneration is reported to be 2.9%, but it can increase to as high as 25.6% after a ten-year follow-up (Hilibrand et al., 1999). Therefore, in order to maintain the range of motion of the operated segment, CDA surgery was first reported in 1990, and a certain range of motion was preserved by inserting an artificial intervertebral disc while decompressing, but the high ossification rate of CDA is also one of its unique disadvantages, and the incidence of heterotopic ossification in two segments undergoing CDA can be as high as 15.9% at 7 years of follow-up (Gornet et al., 2019).

MI-PCF has advantages compared with anterior approaches (ACDF and CDA), such as eliminating the need for internal fixation devices, reducing adjacent segment degeneration, and avoiding pseudoarthrosis formation and heterotopic ossification formation. Posterior minimally invasive surgery avoids dissection of the posterior muscles and avoids anterior approach complications, including the possibility of injuring important esophageal or vascular organs. In North American countries, MI-PCF is less expensive than ACDF, and there is currently little literature to compare the advantages of MI-PCF and anterior approaches (ACDF/CDA). This study compared surgical duration, length of hospital stay, complications, and reoperation rates between MI-PCF and anterior approaches (ACDF/CDA) by systematic review and meta-analysis.

## 2 Methods

### 2.1 Protocol

This study was systematically reviewed following Preferred Reporting Items for Systematic Reviews and Meta-Analysis (PRISMA) statement (Page et al., 2021).

### 2.2 Literature search

PubMed, Cochrane Library, Medline, Embase, and Scopus databases were used to search the literature for comparisons between MI-PCF and ACDF/CDA. Mesh and keywords included: "posterior cervical foraminotomy", "anterior cervical discectomy and fusion", "minimally invasive foraminotomy", "anterior cervical fusion", "posterior foraminotomy", "cervical arthroplasty", and "cervical disc replacement" to identify studies of interest.

### 2.3 Exclusion and inclusion

We conducted a search encompassing publications up to February 2024, including direct comparative studies such as randomized controlled trials, prospective/retrospective cohorts, and case-control studies. Non-English publications, editorial reviews, conference abstracts, book chapters, systematic reviews, meta-analyses, case reports, and case series were excluded. Only clinical studies that directly compared MI-PCF to ACDF/CDA were included in the analysis.

Studies of O-PCF with posterior muscle dissection, anterior cervical foraminotomy studies, and studies of cervical myelopathy were also excluded. Additional manual searches were performed through cited references. The titles and abstracts of relevant articles were initially evaluated independently by two reviewers, and then the full texts of all selected articles were carefully assessed.

### 2.4 Data collection

Studies assessed differences in various indicators, including surgical duration, length of hospital stay, complications, and reoperation rates. A meta-analysis was only performed when three or more studies assessed the same variable. ORs and 95% confidence intervals were calculated for dichotomous outcomes and then pooled by a random-effects model meta-analysis. All statistical tests were performed using RevMan 5.4 software. *I*^*2*^ test was performed to test statistical heterogeneity for each comparison, with *I*^*2*^ values exceeding 25%, 50%, and 75% indicating low, moderate, and high heterogeneity, respectively. *p* < 0.05 was used to assess statistical significance. The quality of each published study included in the meta-analysis was assessed for risk of bias. Specifically, the assessment included selection bias, performance bias, detection bias, attrition bias, and reporting bias.

## 3 Results

### 3.1 Systematic review

A total of 322 abstracts were reviewed, of which 181 were excluded. The full texts of 141 articles were assessed, and 104 of them were subsequently excluded.

**Fig. 1:**
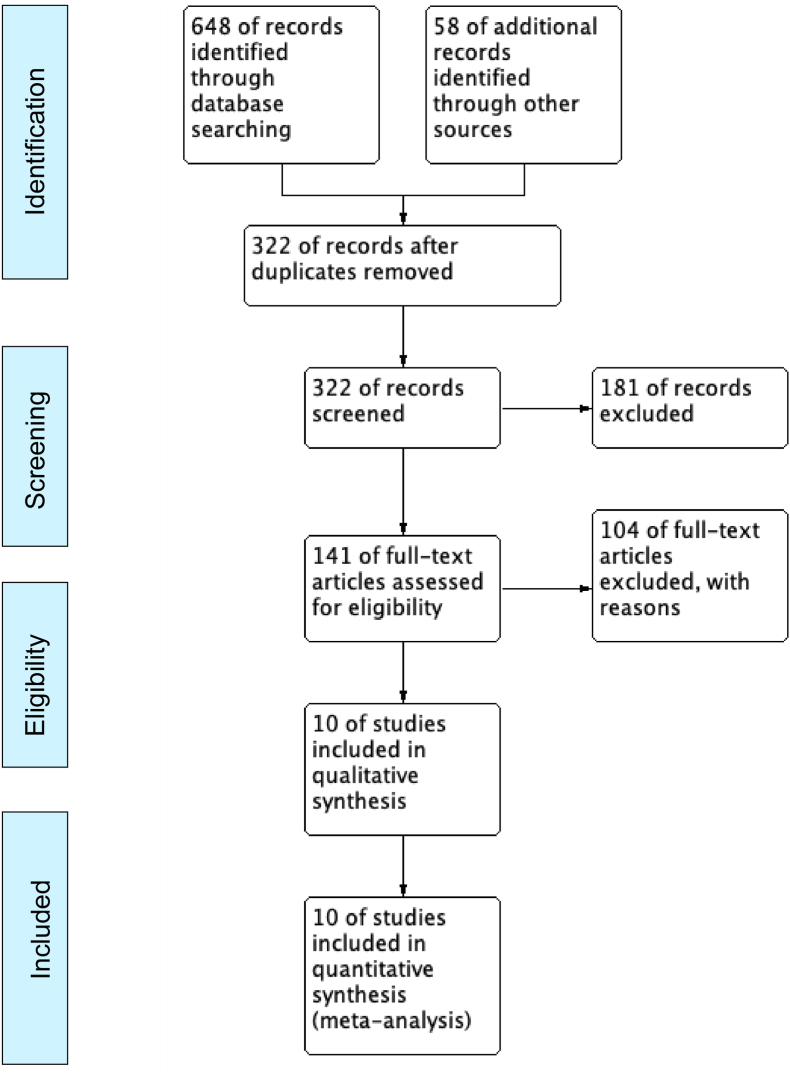
A flow chart of study selections.

For anterior approaches, studies on anterior cervical foraminotomy were excluded, while for posterior approaches, studies on O-PCF procedures and retrospective studies that did not compare posterior cervical foraminotomy with ACDF or CDA were excluded. A total of 10 studies were included, with 6 directly comparing MI-PCF with ACDF (Young et al., 2015; Emami et al., 2022; Ruetten et al., 2008; Dunn et al., 2018; Ji-jun et al., 2020; Mansfield et al., 2014), 2 directly comparing MI-PCF with CDA (Kim et al., 2017; Changoor et al., 2024), and 2 studies comparing MI-PCF with both ACDF and CDA (Lin et al., 2019; Padhye et al., 2022). The study inclusion and exclusion process is illustrated in Fig. 1. The quality of the included studies was independently evaluated by two reviewers.

### 3.2 MI-PCF vs ACDF

**Fig. 2:**
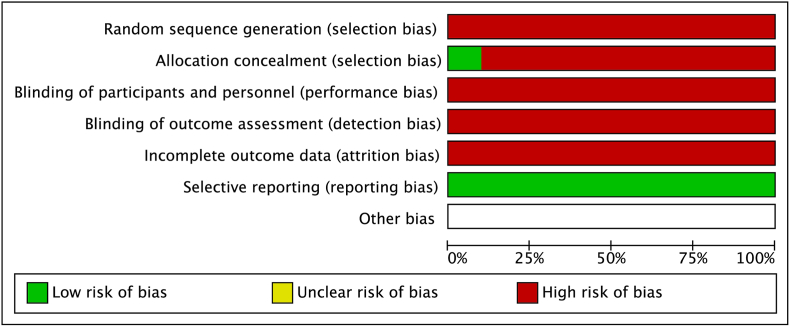
A graphic representation of risk of bias for each included studyFour studies compared the mean.

All 8 included studies met the inclusion criteria and compared the outcomes of patients undergoing MI-PCF and ACDF (Table 1). Among the eight studies, the study by Ruetten et al. (2008) was the only randomized controlled trial comparing the two surgical approaches, while the remaining studies were retrospective cohort series (Young et al., 2015; Emami et al., 2022; Dunn et al., 2018; Ji-jun et al., 2020; Mansfield et al., 2014; Kim et al., 2017; Changoor et al., 2024; Lin et al., 2019; Padhye et al., 2022). Minimally invasive techniques varied across each study, with Ruetten et al. using a 5.9-mm-diameter percutaneous single-port endoscopy (Ruetten et al., 2008). Mansfield et al. (2014) utilized a microscope-assisted approach with a tubular channel. Young et al. (2015) used a self-invented retractor for the posterior keyhole. Dunn et al. (2018) used a 12-mm tubular retractor. Lin et al. (2019) reported the use of minimally invasive techniques, including microscopy with channels and endoscopic techniques. Ji-jun et al.(Ji-jun et al., 2020) used a 5.9-mm-diameter single-port endoscopic technique. Emami et al. (2022) used a microscope with a tunnel technique. Padhye et al. (2022) did not describe their specific minimally invasive approach, but they compared the clinical outcomes of minimally invasive and open PCF separately. All patients were treated for cervical radiculopathy, often caused by cervical disc herniation or foraminal stenosis, which was not distinguished in eight studies. A total of 849 patients were included in the ACDF group and 302 in the MI-PCF group. Each study comparing MI-PCF and ACDF found a high risk of bias (Fig. 2).

**Table 1:** Basic characteristics of included studies comparing MI-PCF to ACDF.

Study	Study type	Country	Level of evidence	Intervention (# of patients)	Mean age (years)	Mean follow-up (months)
Padhye et al., 2022	RCS	USA	III	ACDF (257) MI-PCF (52)	49/54	52/51
Emami et al., 2021	RCS	USA	III	ACDF (205) MI-PCF (43)	50/49.1	98.3/95.9
Ji-jun et al., 2020	RCS	China	III	ACDF (38) MI-PCF (43)	51.4/46.6	NR/NR
Lin et al., 2019	RCS	Korea	III	ACDF (55) MI-PCF (21)	52.5/53.4	39.5/35.9
Dunn et al., 2018	RCS	USA	III	ACDF (210) MI-PCF (49)	49.9/49	44.9/42.9
Young et al., 2015	RCS	USA	III	ACDF (268) MI-PCF (112)	47.4/50.2	141.6/81.6
Mansfield et al., 2014	RCS	USA	III	ACDF (76) MI-PCF (21)	49/49	NR/NR
Ruetten et al., 2008	RCT	Germany	II	ACDF (84) MI-PCF (91)	NR/NR	24/24

surgical duration and found that the mean surgical duration was 59.2–97.8 min in the ACDF group and 28–95.3 min in the MI-PCF group (Table 2). Three studies by Padhye (Padhye et al., 2022), Ruetten (Ruetten et al., 2008), and Ji-jun et al. (Ji-jun et al., 2020). Showed statistically significant differences in surgical duration. In the comparison of postoperative hospital stay between ACDF and MI-PCF groups in the four studies, it ranged from 5.5 to 112.8 h for ACDF, while for MI-PCF, it was 3.8–93.6 h. The findings from the studies by Padhye, Ji-jun, and Mansfield (Ji-jun et al., 2020; Mansfield et al., 2014; Padhye et al., 2022) indicated that this difference was statistically significant.

**Table 2:** Comparison of outcomes following MI-PCF and ACDF.

Study	Intervention (# of patients)	Mean operative time (minutes)	Mean postoperative length of stay (hours)	Complication rate (%)	Reoperation rate (%)
Padhye et al., 2022	ACDF (257) MI-PCF (52)	65/54	34/21	4/2	10/10
Emami et al., 2021	ACDF (205) MI-PCF (43)	NR/NR	NR/NR	2.9/2.2	7.8/8.7
Ji-jun et al., 2020	ACDF (38) MI-PCF (43)	59.2/95.3	5.5/3.8	15.7/4.6	NR/NR
Lin et al., 2019	ACDF (55) MI-PCF (21)	97.8/93.9	112.8/93.6	1.8/14.3	0/14.3
Dunn et al., 2018	ACDF (210) MI-PCF (49)	NR/NR	NR/NR	3.3/0	5.7/8.2
Young et al., 2015	ACDF (268) MI-PCF (112)	NR/NR	NR/NR	NR/NR	2.6/2.7
Mansfield et al., 2014	ACDF (76) MI-PCF (21)	NR/NR	33.84/13.68	NR/NR	1.32/0
Ruetten et al., 2008	ACDF (84) MI-PCF (91)	68/28	NR/NR	6.0/3.3	4.7/6.7

**Fig. 3:**
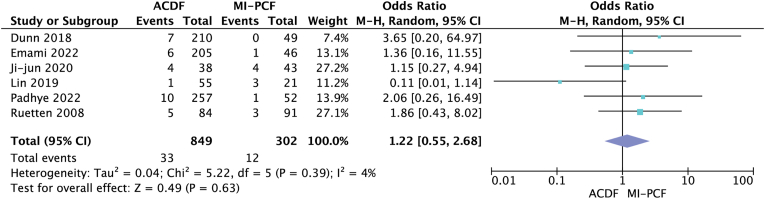
Forest plot comparing complications after ACDF (anterior cervical discectomy and fusion) and MI-PCF (minimally invasive posterior cervical foraminotomy); IV (inverse variance), CI (confidence interval), df (degrees of freedom).

**Fig. 4:**
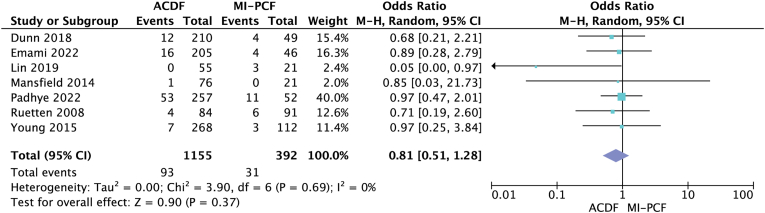
Forest plot comparing reoperations after ACDF (anterior cervical discectomy and fusion) and MIS-PCF (minimally invasive posterior cervical foraminotomy); IV (inverse variance), CI (confidence interval), df (degrees of freedom).

Six studies (Emami et al., 2022; Ruetten et al., 2008; Dunn et al., 2018; Ji-jun et al., 2020; Lin et al., 2019; Padhye et al., 2022) compared postoperative complications and found rates of 1.8%–15.7% in the ACDF group and 0%–14.3% in the MI-PCF group (Table 2). Only one study by Lin et al. (2019) found a statistically significant higher complication rate in the MI-PCF group. The overall complication rate was 3.89% in the ACDF group, and 3.97% in the MI-PCF group, and a random-effects model meta-analysis was performed, which showed no statistically significant difference in complication rates between the two procedures (OR 1.22; 95% CI 0.55, 2.68; p = 0.63, *I*^*2*^ = 4%) (Fig. 3). Seven studies (Young et al., 2015; Emami et al., 2022; Ruetten et al., 2008; Dunn et al., 2018; Mansfield et al., 2014; Lin et al., 2019; Padhye et al., 2022) compared postoperative reoperation rates and found frequencies ranging from 0% to 10% for ACDF and 0%–14.3% for MI-PCF (Table 2). The study by Lin et al. (2019) was the only one to find a statistically significant higher reoperation rate in the MI-PCF group compared to the ACDF group. However, a random-effects model meta-analysis was performed, but failed to show a statistically significant difference in reoperation rates between the two procedures (OR 0.81; 95% CI 0.51, 1.28; p = 0.37, *I*^*2*^ = 0) (Fig. 4).

### 3.3 MI-PCF vs CDA

**Fig. 5:**
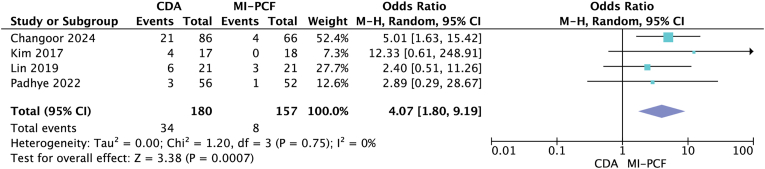
Forest plot comparing complications after CDA (Cervical disc arthroplasty) and MI-PCF (minimally invasive posterior cervical foraminotomy); IV (inverse variance), CI (confidence interval), df (degrees of freedom).

**Fig. 6:**
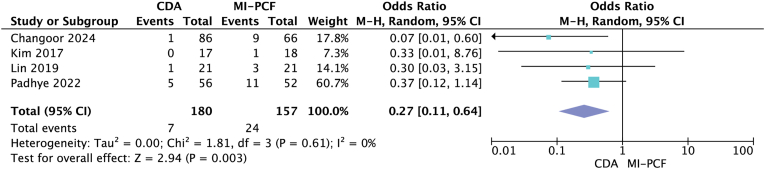
Forest plot comparing complications after CDA (Cervical disc arthroplasty) and MI-PCF (minimally invasive posterior cervical foraminotomy); IV (inverse variance), CI (confidence interval), df (degrees of freedom).

Four studies (Kim et al., 2017; Changoor et al., 2024; Lin et al., 2019; Padhye et al., 2022) that met the inclusion criteria were included, and outcomes between patients undergoing MI-PCF and CDA were directly compared (Table 3). All four studies were retrospective cohort series. Kim et al. (2017) used the Prestige LP artificial disc, and MI-PCF used the METRx system (16- or 18-mm tubular channels). The other three studies (Changoor et al., 2024; Lin et al., 2019; Padhye et al., 2022) did not specify the techniques used. The four studies included CDA patients aged 42.1–52.5 years and MI-PCF patients aged 42.8–55.7 years. The follow-up time ranged from 39.5 to 82.5 months in the CDA group, while it ranged from 35.9 to 84.1 months in the MI-PCF group. All indications were cervical radiculopathy. A total of 180 patients were included in the CDA group, and 157 patients were included in the MI-PCF group. Surgical duration varied from 72 to 106.7 min for CDA and from 54 to 93.9 min for MI-PCF. Kim et al. (2017) showed that the mean surgical duration was statistically significantly shorter in the MI-PCF group. The mean postoperative hospital stay ranged from 26.4 to 165.6 h in the CDA group and from 21 to 98.4 h in the MI-PCF group. Kim et al. (2017) found a statistically significant shorter postoperative hospital stay in the MI-PCF group. The complication rate ranged from 5% to 28.6% in the CDA group, whereas it ranged from 0% to 14.3% in the MI-PCF group (Table 4). Kim et al. (2017) also found a statistically significant decrease in complication rates in the MI-PCF group. The overall complication rate was 18.89% in the CDA group and 5.1% in the MI-PCF group. A random-effects model meta-analysis was performed and showed a statistically significant difference in complication rates between the two procedures (OR 4.07; 95% CI 1.80, 9.19; p = 0.0007, *I*^*2*^ = 0) (Fig. 5). The reoperation rate ranged from 0% to 5% in the CDA group, 5.6%–14.3% in the MI-PCF group. The overall reoperation rate was 3.89% in the CDA group, and 15.29% in the MI-PCF group. Random effects model meta-analysis showed a statistically significant difference in reoperation rates between the two procedures (OR 0.27; 95% CI 0.11, 0.64; p = 0.003, *I*^*2*^ = 0) (Fig. 6).

**Table 3:** Basic characteristics of included studies comparing MI-PCF to CDA.

Study	Study type	Level of evidence	Intervention (# of patients)	Mean age (years)	Mean follow-up (months)
Changoor et al., 2024	RCS	III	CDA (86) MI-PCF (66)	47.2/55.7	39.5/43.1
Padhye et al., 2022	RCS	III	CDA (56) MI-PCF (52)	46/54	42/51
Lin et al., 2019	RCS	III	CDA (21) MI-PCF (21)	52.5/53.4	39.5/35.9
Kim et al., 2017	RCS	III	CDA (17) MI-PCF (18)	42.1/42.8	82.5/84.1

**Table 4:** Comparison of outcomes following MI-PCF and CDA.

Study	Intervention (# of patients)	Mean operative time (minutes)	Mean postoperative length of stay (hours)	Complication rate (%)	Reoperation rate (%)
Changoor et al., 2023	CDA (86) MI-PCF (66)	99.8/79.2	26.4/28.8	24.4/6.2	1.2/13.6
Padhye et al., 2022	CDA (56) MI-PCF (52)	72/54	28/21	5/2	5/10
Lin et al., 2019	CDA (21) MI-PCF (21)	106.7/93.9	103.2/93.6	28.6/14.3	4.8/14.3
Kim et al., 2017	CDA (17) MI-PCF (18)	90.3/77.4	165.6/98.4	23.5/0	0/5.6

## 4 Discussion

With the development of minimally invasive techniques in recent years, MI-PCF has become increasingly popular as a minimally invasive method for the treatment of cervical radiculopathy since it was first proposed in 2000 (Burke; Roh et al., 2000). Unlike Open Posterior Cervical Foraminotomy (O-PCF), which requires extensive dissection of the posterior cervical musculature and often results in postoperative axial pain, MI-PCF effectively circumvents these issues. A previous meta-analysis showed that MI-PCF resulted in reduced blood loss, shorter hospital stay, and less postoperative analgesic use compared with O-PCF (Clark et al., 2011). However, there is a still controversy about whether MI-PCF is superior to anterior approaches (ACDF and CDA) (Zou et al., 2022; Platt et al., 2022). In this review, studies involving treatment cohorts that included O-PCF were excluded. The indications for MI-PCF, ACDF, and CDA are not the same, as ACDF and CDA can be used to treat bilateral foraminal compression (including disc herniation and foraminal stenosis) or central canal lesions. Conversely, MI-CPF is primarily used to treat foraminal compression (including disc herniation and bony stenosis). Thus, all three surgical methods can be suitable for the treatment of cervical radiculopathy with foraminal compression. There is a lack of randomized controlled studies directly comparing MI-PCF techniques with ACDF and CDA with a large sample size with prolonged follow-up, and a lack of comprehensive systematic reviews and meta-analyses. MI-PCF is divided into three techniques: microscopy with tubular access (Adamson, 2001; Fessler and Khoo, 2002; Hilton, 2007), single-port endoscope (Ruetten et al., 2007), and dual-port endoscope (Park et al., 2017; Wang et al., 2023), and there is a lack of literature on direct comparison between MI-PCF with dual-port endoscopy and anterior approaches (ACDF/CDA). Therefore, we classified minimally invasive posterior techniques into one category, and the main indication in the study was cervical radiculopathy.

MI-PCF was not consistently reported as superior to ACDF/CDA in terms of surgical duration. Of the studies that met the inclusion criteria, four (Ruetten et al., 2008; Ji-jun et al., 2020; Mansfield et al., 2014; Lin et al., 2019) compared the mean surgical duration between MI-PCF and ACDF, and four (Kim et al., 2017; Changoor et al., 2024; Lin et al., 2019; Padhye et al., 2022) compared the surgical duration between MI-PCF and CDA. The studies by Padhye, Ruetten, Ji-jun, and Kim et al.(Ruetten et al., 2008; Ji-jun et al., 2020; Kim et al., 2017; Padhye et al., 2022) showed that MI-PCF had shorter surgical duration, and the difference in surgical duration was statistically significant. Ruetten et al. and Ji-jun et al.(Ruetten et al., 2008; Ji-jun et al., 2020)^,20^ used the 5.9 mm-diameter percutaneous single-port endoscopic technique, Kim (Kim et al., 2017) used the METRx system (16- or 18-mm tubular channels), and Padhye (Padhye et al., 2022) did not introduce their minimally invasive approach.

The main differences in time consumption between minimally invasive approaches lie in fluoroscopy and positioning, while for open surgery, the variations are primarily in exposure and suturing. In our study, we did not conduct a meta-analysis on the comparison of surgical duration. MI-PCF is a minimally invasive surgical approach.

In 4 studies (Ruetten et al., 2008; Ji-jun et al., 2020; Mansfield et al., 2014; Padhye et al., 2022), the postoperative hospital stay for the ACDF group ranged from 5.5 to 112.8 h, while for the MI-PCF group, it ranged from 3.8 to 93.6 h. Three studies (Ji-jun et al., 2020; Mansfield et al., 2014; Padhye et al., 2022) by Padhye, Ji-jun, and Mansfield et al. showed that this difference in this regard. Another Four studies (Kim et al., 2017; Changoor et al., 2024; Lin et al., 2019; Padhye et al., 2022) showed a mean postoperative hospital stay of 26.4–165.6 h in the CDA group and 21–98.4 h in the MI-PCF group. Kim et al. (2017) found a statistically significant shorter postoperative hospital stay in the MI-PCF group. However, these results did not clearly demonstrate a reduction in postoperative hospital stay for patients following MI-PCF. Local policies greatly influence the length of hospital stays. Some studies have even conducted these three procedures in outpatient settings to mitigate the costs associated with inpatient wards and care.”Helseth et al. (2019) demonstrated that both MI-PCF and ACDF patients could be discharged on the day of surgery without compromising safety. However, anterior cervical surgery carries the risk of hematoma may slowly accumulate and potentially endanger the patient's life (Song et al., 2017). In particular, in clinical practice,MI-PCF, can be routinely performed in the outpatient clinic without the need for a drainage tube and patients can be discharged on the same day, suggesting that actual average postoperative hospital stay may be much shorter than reported in the above studies.

MI-PCF avoids approach related complications of the anterior approach, but this systematic review and meta-analysis did not show a lower complication rate with MI-PCF. Of the studies that met the inclusion criteria, 6 studies (Emami et al., 2022; Ruetten et al., 2008; Dunn et al., 2018; Ji-jun et al., 2020; Lin et al., 2019; Padhye et al., 2022) compared complication rates between MI-PCF and ACDF, and 4 studies (Kim et al., 2017; Changoor et al., 2024; Lin et al., 2019; Padhye et al., 2022) compared complication rates between MI-PCF and CDA. Lin et al. (2019) found a statistically significant increase in the incidence of complications following MI-PCF compared to ACDF, but unlike other included studies, this study did not describe complications following MI-PCF. The overall complication rate was 3.89% in the ACDF group and 3.97% in the MI-PCF group, and meta-analysis did not show a statistically significant difference in complication rates between the two procedures. In the comparison of MI-PCF compared with CDA, the study by Kim et al. (2017) also found a statistically significant decrease in complication rates in the MI-PCF group. The overall complication rate was 18.89% in the CDA group and 5.1% in the MI-PCF group. A random-effects model meta-analysis was performed and showed a statistically significant difference in complication rates between the two procedures, with a higher overall complication rate for CDA.

Seven studies (Young et al., 2015; Emami et al., 2022; Ruetten et al., 2008; Dunn et al., 2018; Mansfield et al., 2014; Lin et al., 2019; Padhye et al., 2022) compared reoperation rates after ACDF and MI-PCF. Only one study, Lin et al. (2019) found a statistically significant difference between the two, with an increased reoperation rate after MI-PCF. This was not confirmed in the meta-analysis. However, only one of the included studies comparing the results of MI-PCF and ACDF treatment had a follow-up of more than four years. The ACDF case series with long-term follow-up showed that the incidence of adjacent segment disease after surgery increased year by year and could reach up to 25.6% at 10 years (Hilibrand et al., 1999). Future studies with longer follow-up intervals may indicate lower reoperation rates for MI-PCF compared to ACDF. However, reoperation for MI-PCF is primarily due to prominent recurrence and incomplete initial decompression. Given the minimally invasive nature of MI-PCF, the learning curve rules, and the preservation of range of motion, a reduced risk of reoperation for MI-PCF compared to ACDF is anticipated, even lower than for ACDF. The four included studies (Kim et al., 2017; Changoor et al., 2024; Lin et al., 2019; Padhye et al., 2022) compared reoperation rates after MI-PCF and CDA, with an overall reoperation rate of 3.89% in the CDA group and 15.29% in the MI-PCF group. Meta-analysis showed a higher reoperation rate with MI-PCF. This is primarily attributed to the low incidence of reoperation for adjacent vertebral disease in CDA due to the preservation of segmental motion, resulting in a very low overall reoperation rate conversely, MI-PCF, a novel minimally invasive approach, is expected to see a decrease in the proportion of reoperations required due to incomplete decompression as surgeons become more proficient with the technique over time. This is particularly relevant in light of recent reports on the excellent and good rate of the dual-port PCF technique, which may contribute to a lower future reoperation rate for MI-PCF (Wang et al., 2023). Overall, anterior decompression surgeries have broader indications than MI-PCF, as ACDF allows decompression from the central to the medial pedicle walls. In our included studies, all patients with myelopathic conditions were excluded. Both anterior decompression and MI-PCF for cervical radiculopathy compressing the foraminal region can achieve complete decompression.

We have noticed significant discrepancies between the conclusions of some studies and those of this meta-analysis. For instance, Kumar C et al. (Kumar et al., 2019). found the probability of CDA complications to be 1.49%, which is significantly lower than our study, and the probability of reoperation to be 8.06%, which is significantly higher than our study. Upon comparison, we found that this difference is mainly due to different criteria for determining complications: heterotopic ossification, as the most common complication of CDA, does not require special treatment in most cases. Kumar C et al.'s study did not consider heterotopic ossification as a complication, which caused a certain deviation between their conclusions and ours. Therefore, different criteria for determining complications leads to significant differences in the probabilities of complications and reoperation in different studies about CDA.

### 4.1 Limitations

This study has several limitations that warrant discussion. Firstly, the number of included studies was insufficient, with 8 for ACDF and only 4 for CDA. Secondly, although all being direct comparative studies, the results are not uniform and exhibited high heterogeneity, precluding the performance of meta-analysis grading, which limits the generalizability of meta-analysis. This may limit the clarity of our results’ interpretation. Thirdly, the follow-up time and cohort size were also limited and reduced the clinical significance of the statistical differences. Fourthly, the MI-PCF technique used in the various studies varied, particularly the lack of the latest dual-channel MI-PCF. Fifthly, the majority of studies included in the analysis were rated as Level III evidence, retrospective studies, necessitating larger-scale randomized controlled trials in the future to confirm clinical outcomes.

## 5 Conclusion

MIS-PCF and ACDF did not demonstrate a significant advantage in terms of operation time, postoperative hospital stay, complication rate, or reoperation rate. MI-PCF demonstrated a significant advantage over CDA in terms of a lower complication rate, but the reoperation rate of MI-PCF was higher than that of CDA. Further research with longer follow-up time and larger sample size is required to determine the real superiority of MI-PCF in treating cervical spondylotic radiculopathy.

## Ethical approval

Not applicable.

## Consent for publication

Not applicable.

## Funding

This study was supported by grants from the financial support of the National Science Foundation of China (NSFC, 81974349) and 2022 In-Hospital Free Innovation Pre-Research Fund of the Scientific Research Office (F016.01003.22003.138) and Department of Science and Technology of Hubei Province General Foundation of Natural science (2024AFB664).

## Data availability

Not applicable.

## Code availability

Not applicable.

## Consent to participate

Not applicable.

## Consent to publish

Not applicable.

## Conflicts of interest

The author(s) declared no potential conflicts of interest with respect to the research, authorship, and/or publication of this article.

## Author contributions

All authors contributed to the study conception and design. Lin Xie and Hanmo Fang analyzed the data. Min Cui, Kangcheng Zhao and Xianlin Zeng interpreted the patient data. Cao Yang and Lin Xie supervised the review. The first draft of the manuscript was written by Lin Xie and Hanmo Fang, and all authors commented on previous versions of the manuscript. All authors read and approved the final manuscript.
